# Effectiveness of a Web-Based Screening and Fully Automated Brief Motivational Intervention for Adolescent Substance Use: A Randomized Controlled Trial

**DOI:** 10.2196/jmir.4643

**Published:** 2016-05-24

**Authors:** Nicolas Arnaud, Christiane Baldus, Tobias H Elgán, Nina De Paepe, Hanne Tønnesen, Ladislav Csémy, Rainer Thomasius

**Affiliations:** ^1^ German Centre for Addiction Research in Childhood and Adolescence (DZSKJ) Centre for Psychosocial Medicine University Medical Center Hamburg-Eppendorf Hamburg Germany; ^2^ STAD, Centre for Psychiatry Research Department of Clinical Neuroscience, Karolinska Institutet & Stockholm Health Care Services Stockholm County Council Stockholm Sweden; ^3^ VAD, Vereniging voor Alcohol- en andere Drugproblemen Brussels Belgium; ^4^ Clinical Health Promotion Centre Faculty of Medicine Lund University & Skåne University Hospital Malmö Sweden; ^5^ WHO Collaborating Centre Bispebjerg & Frederiksberg Hospital Faculty of Medicine University of Southern Denmark Copenhagen Denmark; ^6^ Centre for Epidemiological and Clinical Research on Addictions National Institute of Mental Health (NIMH) Klecany Czech Republic

**Keywords:** substance use, adolescents, brief intervention, web-based intervention, motivational interviewing, randomized controlled trial

## Abstract

**Background:**

Mid-to-late adolescence is a critical period for initiation of alcohol and drug problems, which can be reduced by targeted brief motivational interventions. Web-based brief interventions have advantages in terms of acceptability and accessibility and have shown significant reductions of substance use among college students. However, the evidence is sparse among adolescents with at-risk use of alcohol and other drugs.

**Objective:**

This study evaluated the effectiveness of a targeted and fully automated Web-based brief motivational intervention with no face-to-face components on substance use among adolescents screened for at-risk substance use in four European countries.

**Methods:**

In an open-access, purely Web-based randomized controlled trial, a convenience sample of adolescents aged 16-18 years from Sweden, Germany, Belgium, and the Czech Republic was recruited using online and offline methods and screened online for at-risk substance use using the CRAFFT (Car, Relax, Alone, Forget, Friends, Trouble) screening instrument. Participants were randomized to a single session brief motivational intervention group or an assessment-only control group but not blinded. Primary outcome was differences in past month drinking measured by a self-reported AUDIT-C-based index score for drinking frequency, quantity, and frequency of binge drinking with measures collected online at baseline and after 3 months. Secondary outcomes were the AUDIT-C-based separate drinking indicators, illegal drug use, and polydrug use. All outcome analyses were conducted with and without Expectation Maximization (EM) imputation of missing follow-up data.

**Results:**

In total, 2673 adolescents were screened and 1449 (54.2%) participants were randomized to the intervention or control group. After 3 months, 211 adolescents (14.5%) provided follow-up data. Compared to the control group, results from linear mixed models revealed significant reductions in self-reported past-month drinking in favor of the intervention group in both the non-imputed (*P*=.010) and the EM-imputed sample (*P*=.022). Secondary analyses revealed a significant effect on drinking frequency (*P*=.037) and frequency of binge drinking (*P*=.044) in the non-imputation-based analyses and drinking quantity (*P*=.021) when missing data were imputed. Analyses for illegal drug use and polydrug use revealed no significant differences between the study groups (*P*s>.05).

**Conclusions:**

Although the study is limited by a large drop-out, significant between-group effects for alcohol use indicate that targeted brief motivational intervention in a fully automated Web-based format can be effective to reduce drinking and lessen existing substance use service barriers for at-risk drinking European adolescents.

**Trial Registration:**

International Standard Randomized Controlled Trial Registry: ISRCTN95538913; http://www.isrctn.com/ISRCTN95538913 (Archived by WebCite at http://www.webcitation.org/6XkuUEwBx)

## Introduction

Early misuse of alcohol and other drugs is widespread in Europe with higher prevalence compared to other regions in the world such as the United States [1, 2]. Although temporary substance misuse is a common and partly normative phenomenon in youth development [3,4], adolescence is a critical period for the development of addiction problems. This period is typical for initiation, and rapid escalation of individual problematic substance use patterns into clinically significant problems can be observed among a substantial proportion of youth in Europe [5, 6]. Early excessive drinking and combined use of alcohol with other psychoactive substances (ie, polydrug use) are of particular relevance [7-11] due to the associated adverse effects on physical, psychological, and social functioning that put youth at a heightened risk for long-lasting disadvantages [12-14].

The widespread use of alcohol and other drugs suggests that current capacities to prevent youth from initiating alcohol and other drug use are limited [15, 16]. Prevention efforts should therefore target at-risk youth with indicated preventive interventions [17, 18]. Effective methods to prevent risky substance use and addiction problems are in principle available, but existing health service provision is limited in accessibility and acceptability [19, 20] with the result that interventions are often provided too late and do not reach the majority of at-risk subjects [21, 22].

Web-based intervention programs have been increasingly acknowledged in their capacity to lessen existing service barriers particularly for at-risk populations [23-25]. Moreover, fully automatic delivery (ie, stand-alone or self-guided with no clinician involvement) allow for standardized delivery and can be disseminated cost-effectively at a large scale [24]. Due to the high Internet access rates in contemporary societies and the fact that youth typically use the Internet when searching for information about alcohol and drugs and also are reluctant to disclose alcohol- and drug-related behavior in face-to-face contacts, Web-based interventions hold promise for younger populations [26, 27]. Evidence indicates that fully automated brief motivational interventions can reduce drinking and related harms for emerging adult at-risk drinkers up to 12 months after the intervention [28-31].

The literature on Web-based interventions for illegal drug use is not as developed as it is for alcohol, but a recent meta-analysis (including 10 studies) suggests that overall the effects are somewhat smaller (*g*=0.16) compared to drinking (*g*=0.20-0.39) but significant and (as for alcohol interventions) independent of intervention venue (home vs research setting) and level of guidance through the intervention [32-34].

Although previous studies that have proven the usefulness of Web-based motivational interventions to address substance use and related problems mainly targeted emerging adults [35], the motivational methods that have been studied and found effective are relevant for many risk factors in adolescence, such as their susceptibility to peer influences [36-39]. Motivational interventions are based on the therapeutic style and techniques put forward by Motivational Interviewing (MI) [40], which makes a strong case for conceptual compatibility with adolescent-specific needs for autonomy, subjective perceptions of invulnerability to apparent health risks, and appraisal for short-term benefits at the cost of possible long-term adverse effects [17, 37, 38]. Although effects of MI interventions for alcohol misuse and applied techniques in brief interventions such as normative feedback have recently been summarized as rather small [41, 42], relevant previous studies have shown favorable results from face-to-face [43, 44] as well as Web-based MI-interventions among young adults [29-31]. However, studies targeting mid-to-late adolescents (aged 16-18 years) are lacking [45] despite the fact that this is a critical period for establishing problematic alcohol and other drug use [6, 46] and rapid acceleration for first use of illegal drugs [47, 48].

The purpose of this study was therefore to test the effectiveness of a fully automated Web-based brief MI in a sample of at-risk substance-using adolescents in four European countries. Our primary hypothesis was that participants in the intervention group would report significantly lower levels of past-month drinking (frequency, quantity, and frequency of binge drinking) at 3-month follow-up relative to baseline when compared to an assessment-only control group. Additional hypotheses concerned differences in past-month illegal drug use and combined use of alcohol and illegal drugs.

## Methods

**Figure 1:**
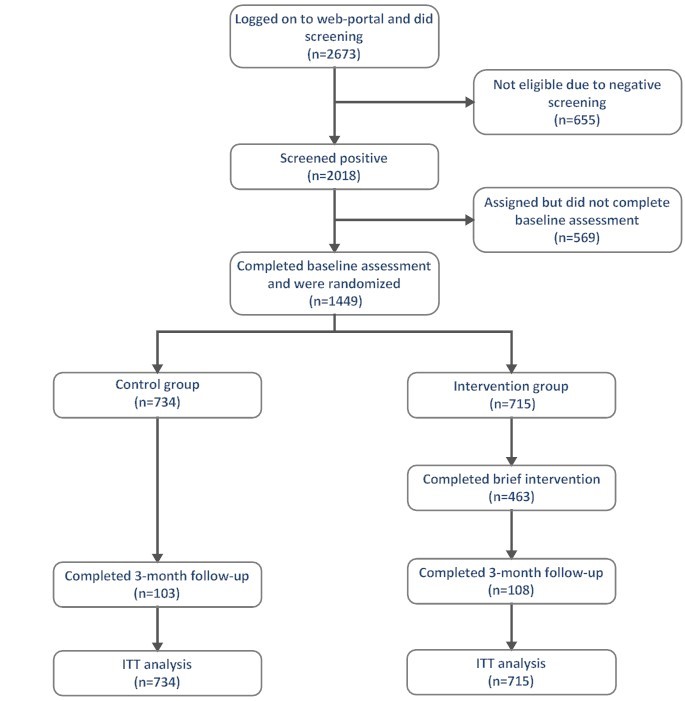
Participant flow.

A two-armed multisite randomized controlled trial (RCT) design was applied in Sweden, Belgium, the Czech Republic, and Germany. Inclusion criteria were being 16-18 years old, online access, informed consent, and a positive screening for at-risk substance use. Baseline assessment was collected at study entry and a follow-up assessment was collected 3 months after baseline assessment. Figure 1 displays the trial design (see Multimedia Appendix 1[49] for the CONSORT EHEALTH checklist).

**Figure 2:**
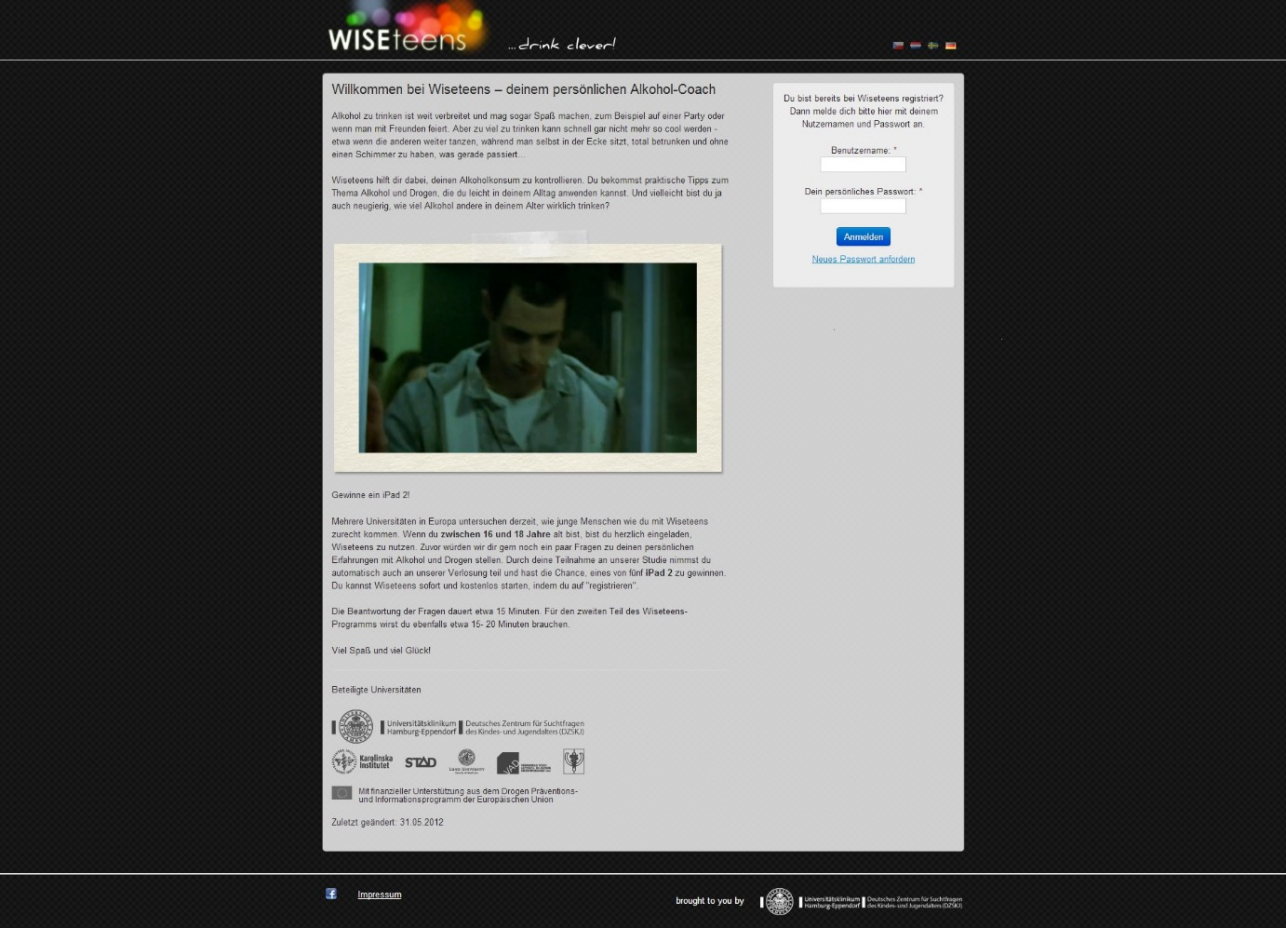
WISEteens landing page.

Five university research centers in Europe developed the purely Web-based content of the WISEteens portal between June 2011 and March 2012. The IT platform was established together with GAIA AG, Hamburg. The landing page (see Figure 2) was designed to create an appealing first impression using visual material (eg, pictures, video). It described the main features of the study by highlighting confidentiality, content and source credibility, and provided a brief guided enrollment procedure [50]. Key to developing the content was the integration of MI principles and techniques in a single session together with an open-access delivery format applying a design to match end-user characteristics and preferences. Furthermore, the content should be acceptable, easy to use, and perceived as relevant by the target group [50-53].

All material was developed in a multidisciplinary team including experts in clinical health promotion, developmental and clinical psychology, and certified behavioral therapists. It was first developed in English and then translated by professional offices into the respective countries’ languages. The Web portal was simultaneously launched in all four countries in June 2012 with recruitment until March 2013. Ethical approval was granted by the responsible Ethics Committees in all participating countries: Chamber of Physicians Hamburg (Germany), Prague Psychiatric Centre (Czech Republic), University Hospital of Antwerp and the University of Antwerp (Belgium), and the Regional Ethics Board in Stockholm (Sweden). The trial design was published [54], and the study was registered in a public database. No content or methodological modifications were made after trial commencement.

### Recruitment

We promoted the open-access WISEteens landing page to recruit a convenience sample of potential participants using both online and offline strategies. As offline strategies, we developed print promotion materials (information leaflets and flyer cards) and distributed them in schools, youth-clubs, cafés, bars, stores, and adolescent specific events. We also used a three-fold online recruitment strategy with high rank of our websites’ domain in widely used search engines, advertisements via popular social media, and links on affiliated health promotion sites. To motivate study participants and enhance follow-up rates, we promoted and held a prize draw for tablet computers among participants who provided follow-up assessment.

### Procedure and Randomization

The participants were anonymous throughout the study. At the first visit, they were asked to register, which required a user name, email address, and a password that did not contain their name. On the landing page, they could choose their respective language flag for a different language than pre-defined by browser options. After registration, respondents were screened for at-risk substance use. Those fulfilling the inclusion criteria then received study information including confidentiality, voluntariness of participation, and data security, as well as information about the randomization protocol. Participants were not blinded to random allocation. After informed online consent, the baseline assessment was completed, including those items that form the base for tailoring intervention content. Participants in the intervention condition received a login code to enable exit and re-entrance. Randomization was generated automatically by an online computer program without stratification. The envisioned number of participants was sufficient to ensure randomization integrity and a likely balanced distribution among the two parallel groups [55].

### Screening

An adapted version of the 6-item CRAFFT (Car, Relax, Alone, Forget, Friends, Trouble) tool was used to screen for at-risk use of alcohol and other drugs (see Multimedia Appendix 2). This tool has proven criterion validity compared to other screening tools [56] and is recommended for identification of at-risk adolescents [17]. A CRAFFT score with at least two positive items was the criterion for study inclusion [57].

### Intervention

The WISEteens intervention relied on an interactive system to generate individually tailored content. All system-generated information was presented in small units that combined text and graphics (eg, photos and illustrative drawings) and directly referred to the participant’s statements assessed in the first place (eg, substance use, sex, weight, perceptions of normative drinking). Navigation through the program was designed as a dialogue between the user and a virtual expert with “gates” (ie, choice options) at the end of each page to permit varying degrees of approval or disapproval with page content. The system used these responses to introduce subsequent content on the next page.

Intervention content, dialogue tone, and style was based on MI [40] and consisted of the following 6 components (see Multimedia Appendix 3 for screenshots), with the first three applying to alcohol but not illegal drug use and polydrug use: (1) feedback for individual drinking patterns with information on associated health and developmental risks, (2) normative feedback to descriptive drinking norms about sex- and age-matched peer drinking levels using graphed comparative information, (3) feedback for blood alcohol concentration (BAC) and associated health and other risks (ie, traffic crash, unintended sex) for the peak drinking episode in the last 2 weeks, (4) importance and confidence rulers with a short summary and feedback to encourage change readiness and exploration of personal strengths, resources, and volitional strategies for goal attainment, (5) decisional balance for selection of personal costs and benefits of current substance use and a subsequent graphical display of comparative gains and losses of behavior change in a balance sheet to illustrate ambivalence, and (6) identification and selection of personal high-risk situations for substance use and provision of behavioral strategies, for example, to resist peer pressure (the assumed mechanisms for change are displayed in Multimedia Appendix 4; for a more detailed description, see [54]).

The WISEteens intervention was pilot-tested in two steps. First, 10 adolescents chose their preferred design concept among three options. A preliminary version with the preferred “look & feel” was then pre-tested by 37 other adolescents to ensure ease of registration and navigation use, comprehensibility of intervention content, satisfaction with layout and design, appropriateness of dialogue style (eg, avoiding judgmental and confronting language), overall satisfaction with the program, and time to complete baseline assessment and intervention. Furthermore, open feedback, technical problems, translational ambiguities, and other problems were documented and the program was adapted accordingly. Median for assessment and intervention was 15 minutes, ranging from 5-30 minutes. Most adolescents were satisfied or totally satisfied with the system, design, comprehensibility, and intervention dialogue. Table 1 provides a summary of the pilot-results. The control group received assessment only and was directed to a short information page on where to find help in case of urgent counseling or medical needs.

**Table 1:** Results pilot-test (N=37^a^).

Items		Response					
		Mean (SD)	Yes, %	Neutral, %	No, %	Median	Range
Overall satisfaction with the program^b^ (1=“not satisfied at all”, 4=“totally satisfied”)		3.1 (0.50)					
Acceptance of program layout and design^c^ (1=“not satisfied at all”, 4=“totally satisfied”)		3.2 (0.66)					
Program comprehensibility^d^ (1=“not satisfied at all”, 4=“totally satisfied”)		2.9 (0.80)					
**Acceptance of dialogue style and tone**							
	“preachy”		0	43.8	56.3		
	“non-judgmental”		68.8	12.5	18.8		
	“appropriate”		81.3	12.5	6.3		
Duration to complete baseline assessment		16.5 min (5.5)				15 min	10-25 min
Duration to complete intervention		15.5 min (7.1)				15 min	5-30 min

^a^Mean age 16.38 (SD 1.23) years; 81.3% men.

^b^Single item on overall satisfaction.

^c^9 items (eg, login/registration procedure, clarity/structure, text amount, graphic design; Cronbach α=.89).

^d^7 items (eg, content comprehensibility, response format, information amount; Cronbach α=.77).

### Data Collection and Measures

All study measures were administered anonymously and online via self-reports at baseline (t_0_ before randomization) and at follow-up (t_1_, 3 months after t_0_) and required registration with a valid email address. Three months after completing the baseline assessment, participants were automatically invited to participate in the follow-up assessment and guided by an integrated hyperlink in the email invitation with one reminder email after 1 week.

Participants were asked for age, gender, country of residence (Sweden, Germany, Belgium, Czech Republic or other), current school attendance (yes/no), parental educational attainment (low, middle, high), their weight in kilograms for BAC-level feedback, descriptive norms [58, 59], and 12-months scores on the Alcohol Use Disorder Identification Test Consumption subscale (AUDIT-C [60]). To address baseline change motivation as a potential confounder, we included intention to reduce drinking (in the next 30 days) using a single item (“I intend to reduce my drinking during the next 30 days”, 1=“totally disagree” to 7=”totally agree”) and intention to abstain from illegal drug use (“I intend to abstain from taking illegal drugs during the next 30 days”, 1=“totally disagree” to 4=“totally agree”) [57], as a continuous measure for change readiness [61].

### Substance Use

All outcome measures concerned use in the past 30 days. Change in alcohol use (frequency, frequency of binge drinking, and quantity) between the two assessments was the primary outcome and measured based on the three items of the AUDIT-C screening tool [60]. This measure provides a widely used and valid index sum score for problem alcohol use of adolescents [62]. The three indicators are drinking frequency (“How often did you have a drink containing alcohol?”; 0=“never” to 4=“four or more times a week *”*), binge drinking frequency (“How often did you have 5 [4 for girls] or more drinks on one occasion, like during a party or on one night?”; 0=“never” to 4=“four or more times a week”), and drinking quantity (“How many drinks containing alcohol did you have on a typical day when you were drinking?”; 0=“one or two” to 4=“ten or more”). To assess the number of consumed drinks, we used a graphical display of various types of drinks with the indication to select the number of each drink per typical drinking occasion to account for national differences in typical standard drinks. Standard drinks were overall defined as containing 10-12 grams of pure ethanol, and responses were recoded to match the original 0-4 point scale. Using an index for drinking has two advantages over separate measures of alcohol use. First, it allows for modeling one drinking measure to increase the statistical power to detect intervention effects while maintaining several indicators of risky drinking. Second, due to the scale, characteristic outcome data can be analyzed as continuous data, which makes interpretation easier compared to discrete count drinking outcomes [63, 64]. We included the three separate outcomes (frequency, frequency of binge drinking, and quantity) and frequency of illegal drug use (1=“never” to 5=“four or more times a week” [65]) as secondary outcomes and past 30 days prevalence of illegal drug use and polydrug use as additional secondary outcomes. Because most drug use combinations in Europe include alcohol [10], we defined polydrug use as a dichotomous measure for use of alcohol and any illegal drug during the last 30 days, similar to previous studies [9].

### Sample Size

Sample size calculation was based on the primary outcome with regard to effect sizes for alcohol use revealed by a recent review on Web-based interventions for young people [31]. According to results from similar studies, we expected a small effect size (Cohen’s *d*=0.2). To reach power of 80% at a type I error rate of 5% in a two-sided test and expecting a dropout rate of approximately 50% [27, 66], we aimed at N=400 per intervention condition [67]. Possible country dependent clustering effects were not included in the sample size calculation because the study was designed as an individual-based RCT and higher level effects from four clusters were not previsioned [68]. Nevertheless, possible higher order country effects were addressed in all further analyses as described below.

### Statistical Analyses

We first analyzed data on sample characteristics using *t*-tests (for metric data) and chi-square tests (for categorical data) to test for differences between intervention conditions. Next, we performed logistic regressions with completers (ie, those who provided valid follow-up data) versus dropouts as the binary dependent variable to test for possible attrition bias using all available sociodemographic and substance use variables as predictors. Intervention effects for primary and continuous secondary outcomes were tested using Linear Mixed Models (LMM) and binary secondary outcomes were analyzed using Generalized Linear Mixed Models. In all LMMs, we used change in outcome scores from baseline as the dependent variable, intervention condition as the only independent variable, baseline values as covariates (“fixed effects”), and country of residence as a single random effect (“random intercept”). This model controls for the correlation between baseline and follow-up outcome scores and does not require a repeated statement (ie, “time”) and no time x group interaction term to interpret intervention effects. Because we found no higher order effect for country of residence (primary outcome: Wald Z=.77, *P*=.441), we skipped the random effect and adjusted the analyses for country of residence and possible confounders (ie, variables that were not balanced between intervention and control group at baseline) and predictors for missing data as additional covariates, which resulted in improved model fit (delta Bayesian information criterion=-13.16) (see [69] for a similar approach). Binary secondary outcome analyses (prevalence illegal drug use and polydrug use) focused on follow-up outcome values rather than change scores. All analyses are based on a complete-case dataset and an intention-to-treat (ITT) sample with imputation of missing follow-up data based on expectation maximization (EM). Both results are relevant and commonly reported in Web-based interventions particularly when dropout is large [70]. EM is a single imputation method that was shown to outperform the multiple imputation module available in SPSS in eHealth studies with high dropout rates [66]. Given the huge dropout in this study, we cross-checked the ITT-outcome analysis using a full information maximum likelihood (FIML) estimator for missing follow-up data provided in the structural equation model software AMOS to reduce estimate bias for missing data [71] and increase the robustness of findings (see Multimedia Appendix 5). For all analyses, we report estimated marginal means (percentages) and Cohen’s *d* effect sizes. Distributions of outcomes (ie, skew and kurtosis) and missing-at-random requirements of missing data were checked prior to the main analyses. Results with a type I error rate of *P*<.05 in 2-sided tests were considered as statistically significant without adjustment for multiple comparisons but reporting of exact *P*values [72]. All analyses other than in AMOS were performed using SPSS statistical software package version 22 [73].

## Results

### Sample Characteristics and Preliminary Analyses

The trial profile is shown in Figure 1. A total of 2673 participants logged on the WISEteens Web portal and participated in the initial screening. We excluded 655 (24.5%) from the study due to a negative CRAFFT screening. This resulted in 2018 (75.5%) adolescents who gave consent to participate in the study and started subsequent baseline assessment (t_0_). A total of 569 (28.1%) dropped out during the baseline assessment leaving 1449 participants who completed baseline assessment and were randomized to either the intervention (N=715) or control group (N=734). In the intervention group, 453 (63.4%) completed the brief intervention as measured by a log file record whether the last page of the intervention has been visible to the user. A total of 211 adolescents participated in the follow-up assessment after 3 months, corresponding to a valid response rate of 14.5%. In this subsample, the completion rate for the brief intervention was higher than in the full randomized sample (82.4%).

In the randomized sample (ITT population), the mean age was 16.8 years (SD 0.74), nearly half of the participants were women and the majority were currently attending school. Most participants were recruited in the Czech Republic due to a more intense offline recruitment in this country, indicating that adjustment of country of residence as an additional covariate was required in subsequent analyses. Participants in the intervention group tended to have a higher rate of parental educational attainment compared to the control group as indicated by a near significant difference (*P*=.060). This variable was therefore adjusted as an additional covariate in subsequent analyses [68]. With the exception of binge drinking frequency (*P*=.048), there were no significant group differences with regard to demographic or assessment data at baseline (see Table 2). Importantly, there were no baseline differences in the intention to change current alcohol and illegal drug use among the groups. Distributions of all continuous outcome variables showed acceptable skew and kurtosis with values well below 1.0 for baseline and follow-up assessments [74]. Regarding characteristics of participants who provided data at follow-up, group comparisons revealed no significant differences in any assessed variable (see Table 3). Overall baseline group comparisons thus indicate that the randomization was successful and that the completer-only subsample appears largely similar to the randomized sample.

Response rates were very similar for the intervention group (15%) and the control group (14%). Logistic regression analyses with attrition at follow-up as the dependent variable and all demographic (country, parental educational attainment, sex, age) and substance use‒related variables (all primary and secondary outcomes) as predictors explained 8.4% of the total response variance (Nagelkerke’s R^2^). Corresponding odds ratios (OR) revealed country of residence as the only significant predictors for dropout. Response rates were significantly lower for participants from the Czech Republic (11%) than for those from Sweden (23%, 58/251; OR 2.42, 95% CI 1.52-3.85, *P*<.001) and Germany (27%, 31/146; OR 2.514, 95% CI 1.41-4.49; *P*<.001) but not Belgium (18%, 26/143; OR 1.46, 95% CI 0.76-2.80; *P*=.26). The analysis thus indicates no biased attrition based on the available variables except for country of residence, which was adjusted in all subsequent analyses as a relevant predictor of missing data [75]. Analogous attrition analyses for intervention completion (as indicated by a record whether a user has “seen” the last page of the intervention dialogue) indicated that 12.2% of the total response variance was predicted by all study variables, with significantly more completers being women (*P*=.007) and significant differences between participants depending on country of residence (*P*<.001) with the highest intervention completion in Germany (89.7%), followed by Belgium (71.4%), Sweden (69.8), and the Czech Republic (56.0%).

**Table 2:** Baseline values for participant demographic and substance use‒related variables by intervention condition (randomized sample N=1449).

		Intervention	Control	*P*^a^
Randomized sample, n^b^ (%)		715 (49.3)	734 (50.6)	
Age in years, mean (SD)		16.81 (0.75)	16.85 (0.74)	.253
Sex (women), %		47.8	48.6	.758
**Country of residence, %**				.574
	Sweden	16.2	18.4	
	Germany	10.9	9.3	
	Belgium	9.8	9.9	
	Czech Republic	63.3	62.4	
School status (yes), %		95.0	94.8	.885
**Parental education level**^c^**, %**				.060
	Low	10.1	10.9	
	Middle	61.4	66.3	
	High	28.5	22.9	
Intention to reduce alcohol^d^, mean (SD)		3.04 (2.30)	3.22 (2.31)	.136
Intention to abstain from illegal drugs^d^, mean (SD)		5.31 (2.46)	5.40 (2.40)	.501
Descriptive peer drinking norms, mean (SD)		2.31 (0.79)	2.32 (0.78)	.714
Substance use related risk (CRAFFT sum score), mean (SD)		2.75 (1.42)	2.72 (1.35)	.608
Age at first alcohol use, mean (SD)		12.92 (2.30)	13.00 (2.20)	.497
Alcohol use^e^ (last 12 months), mean (SD)		4.91 (2.49)	5.10 (2.58)	.167
Alcohol use^f^ (last 30 days), mean (SD)		5.43 (2.74)	5.46 (2.82)	.803
Drinking frequency^g^, mean (SD)		2.01 (0.84)	1.97 (0.89)	.413
Drinking quantity^g^, mean (SD)		1.79 (1.45)	1.84 (1.48)	.586
Binge drinking frequency^g^, mean (SD)		1.67 (0.92)	1.78 (0.91)	.048
Illegal drug use (last 30 days), %		45.0	43.0	.460
Polydrug use (last 30 days), %		49.2	40.1	.734

^a^Results of chi-square tests for categorical and *t*tests for continuous measures.

^b^May differ for individual variables due to single missing values.

^c^Father’s highest educational attainment.

^d^Scores ranging from 1-7 with higher scores indicating higher motivation for change.

^e^AUDIT-C index score, past 12 months.

^f^AUDIT-C based index score, past 30 days (primary outcome).

^g^Separate drinking indicators, scores ranging from 0-4 with higher scores indicating more severe drinking.

**Table 3:** Baseline values for participant demographic and substance use‒related variables by intervention condition (completers-only sample N=211).

		Intervention	Control	*P*^a^
Completers-only sample, n^b^ (%)		108 (51.2)	103 (48.8)	
Age in years, mean (SD)		16.87 (0.71)	17.03 (0.76)	.130
Sex (women), %		52.9	52.6	.959
**Country of residence, %**				.955
	Sweden	26.9	28.2	
	Germany	15.7	13.6	
	Belgium	13.0	11.7	
	Czech Republic	44.4	46.6	
School status (yes)		88.0	88.3	.961
**Parental education level**^c^**, %**				.198
	Low	14.6	12.4	
	Middle	43.8	57.3	
	High	41.6	30.3	
Intention to reduce alcohol^d^, mean (SD)		2.76 (2.12)	3.13 (2.08)	.223
Intention to abstain from illegal drugs^d^, mean (SD)		5.39 (2.37)	5.59 (2.22)	.539
Descriptive peer drinking norms, mean (SD)		2.33 (0.66)	2.35 (0.64)	.856
Substance use related risk (CRAFFT sumscore), mean (SD)		2.56 (1.32)	2.80 (1.35)	.210
Age at first alcohol use, mean (SD)		12.97 (2.34)	13.06 (2.27)	.781
Alcohol use^e^ (last 12 months), mean (SD)		4.89 (2.27)	5.20 (2.47)	.348
Alcohol use^f^ (last 30 days), mean (SD)		5.35 (2.44)	5.53 (2.87)	.644
Drinking frequency^g^, mean (SD)		2.09 (0.79)	2.04 (0.89)	.695
Drinking quantity^g^, mean (SD)		1.77 (1.27)	1.85 (1.46)	.694
Binge drinking frequency^g^, mean (SD)		1.54 (0.86)	1.69 (0.93)	.261
Illegal drug use (last 30 days), %		42.6	39.5	.946
Polydrug use (last 30 days), %		37.0	31.1	.313

^a^Results of chi-square tests for categorical and *t*tests for continuous measures.

^b^May differ for individual variables due to single missing values.

^c^Father’s highest educational attainment.

^d^Scores ranging from 1-7 with higher scores indicating higher motivation for change.

^e^AUDIT-C index score, past 12 months.

^f^AUDIT-C based drinking index score, past 30 days (primary outcome).

^g^Separate drinking indicators, scores ranging from 0-4 with higher scores indicating more severe drinking.

### Primary Outcome: Past Month Drinking Index

Tables 4 and 5 report the primary and secondary intervention outcomes of this trial at follow-up based on the non-imputed completer-sample and Tables 6 and 7 for the EM-imputed intention-to-treat-sample. All analyses concern substance use in the past 30 days and were adjusted for baseline scores, country of residence, and parental educational attainment.

Based on the non-imputed sample 3 months after the intervention, participants in the intervention group have reduced their drinking as indicated by reduced AUDIT-C based scores relative to baseline with an adjusted mean change of -0.85 (95% CI -1.49 to -0.26) while those in the control group slightly increased their drinking as indicated by a mean increase in drinking of 0.16 (95% CI -0.50 to 0.82). Adjusted mean differences between both groups were 1.02 (95% CI 0.25-1.79) and statistically significant (*F*_1,134_=6.8, *P*=.010, *d*=.26). The corresponding between-group effect was smaller in the (imputed) ITT analysis due to significant reductions relative to baseline in the control group. However, the significant between-group effect (0.16, 95% CI 0.02-0.25) was maintained (*F*_1,1329_=5.2, *P*=.022, *d*=.04). Additional analysis in AMOS based on an FIML estimation for missing outcome assessments confirmed these results (B=-0.72, β=-0.13, *P*=.046; see Multimedia Appendix 5).

### Secondary Outcomes: Drinking (AUDIT-C‒Based Separate Items)

We conducted identical analyses for the three drinking indicators separately. In the non-imputed sample, we found a significant between-group difference in drinking frequency of 0.25 (95% CI 0.02-0.50) in favor of the WISEteens group (*F*_1,134_=4.4, *P*=.037, *d*=.15), which was not maintained in the analysis based on EM-imputation (*F*_1,1329_=3.2, *P*=.073, *d*=.11) due to significant reductions relative to baseline in both groups (*P*s<.001). We obtained a similar result for binge drinking frequency with a significant adjusted mean difference between groups of 0.31 (95% CI 0.01-0.61; *F*_1,121_=4.2, *P*=.044, *d*=.16) in the non-imputed data analysis, which was not maintained in the imputed analysis (*F*_1,1329_=2.3, *P*=130, *d*=.01). Additional FIML analysis for both outcomes revealed non-significant and similar results as in the imputed analysis (drinking frequency: B=-0.13, β=-0.08, *P*=.230 and binge drinking frequency: B=-0.25, β=-0.14, *P*=.059; see Multimedia Appendix 5). For drinking quantity, we found significant reductions relative to baseline for the intervention group in the non-imputed analysis of -0.39 (95% CI -0.72 to -0.06, *P*=.024), but these reductions were non-significantly different from the control group (between-group differences: 0.31, 95% CI -0.17 to 0.62; *F*_1,155_=1.3, *P*=.257, *d*=.13). This effect was significant in the EM-imputed data-set (*F*_1,1329_=5.3, *P*=.021, *d*=.05). However, when cross-checked using the FIML approach employed in AMOS, these differences were no longer significant (B=-0.22, β=-0.08, *P*=.209, see Multimedia Appendix 5).

### Secondary Outcomes: Illegal Drug Use and Polydrug Use

Results for frequency and prevalence of illegal drug use as well as polydrug use prevalence are summarized in Tables 5 and 7. With regard to these outcomes, we found no statistically significant between-group effects in the non-imputed (*P*s=.138-.311) and the EM-imputed (*P*s=.363-.871) datasets. Although overall, both groups show numerical decreases between the measurements that were statistically significant in the intervention group for illegal drug use prevalence (*P*=.025) and polydrug use prevalence (*P*=.012) in the non-imputed analyses and statistically significant for all 3 outcomes (ie, frequency of illegal drug use and illegal drug use and polydrug prevalence, *P*s<.001) in the imputed analyses.

**Table 4:** Intervention effects^a^ on primary and (continuous) secondary outcomes (non-imputed dataset^b^).

Outcomes after 3 months		WISEteens group (n=715)			Control group (n=734)			Between-group differences			
		Mean (SD)	Change from baseline, adjusted mean (95% CI)	*P*	Mean (SD)	Change from baseline, adjusted mean (95% CI)	*P*	Adjusted mean (95% CI)	*F*(df)	*P*	*d*
**Index alcohol use**^c^											
	Baseline	5.43 (2.74)			5.46 (2.81)						
	3-months follow-up	4.59 (2.77)	-0.85 (-1.49 to -0.26)	.009	5.35 (2.57)	0.16 (-0.50 to 0.82)	.614	1.02 (0.25 to 1.79)	6.80 (1, 134)	.010	.26
**Drinking frequency**^d^											
	Baseline	2.01 (0.84)			1.97 (.90)						
	3-months follow-up	1.80 (0.84)	-0.36 (-0.55 to -0.16)	<.001	1.88 (0.81)	-0.11 (-0.31 to 0.10)	.305	0.25 (0.02 to 0.50)	4.40 (1, 144)	.037	.15
**Binge drinking frequency**^d^											
	Baseline	1.67 (0.92)			1.78 (0.91)						
	3-months follow-up	1.39 (0.95)	-0.11 (-0.36 to 0.14)	.375	1.66 (0.85)	0.20 (-0.07 to 0.47)	.152	0.31 (0.01 to 0.61)	4.20 (1, 121)	.044	.16
**Drinking quantity**^d^											
	Baseline	1.79 (1.45)			1.84 (1.48)						
	3-months follow-up	1.59 (1.39)	-0.39 (-0.72 to -0.06)	.024	1.83 (1.36)	-0.16 (-0.50 to 0.17)	.336	0.23 (-0.17 to 0.62)	1.30 (1, 155)	.257	.13
**Illegal drug use frequency**^e^											
	Baseline	0.87 (1.20)			0.80 (1.14)						
	3-months follow-up	0.69 (1.10)	-0.13 (-0.37 to 0.11)	.292	0.71 (1.07)	-0.03 (-0.28 to 0.22)	.805	0.10 (-0.21 to 0.40)	0.40 (1, 133)	.532	.07

^a^Based on linear mixed model with group as fixed factor, changes from baseline as outcomes, and baseline scores, country, and parental educational attainment as covariates for continuous outcomes. Cohen’s *d* calculated by subtracting the average difference score between pretest and posttest of the control group from the corresponding difference score of the intervention group, and dividing the result by the pooled standard deviation of the baseline scores.

^b^Valid follow-up data for n=211 trial participants.

^c^Adapted AUDIT-C index score (primary outcome).

^d^Adapted AUDIT-C indicators, scores ranging from 0-4 with higher scores indicating more severe drinking.

^e^Scores ranging from 0-4 with higher scores indicating more frequent illegal drug use.

**Table 5:** Intervention effects^a^ on binary secondary outcomes (non-imputed dataset^b^).

Outcome after 3 months		WISEteens group (n=715)		Control group (n=734)		Between-group differences		
		% (SE)	*P*	% (SE)	*P*	*F*(df)	*P*	OR (95% CI)
**Illegal drug use prevalence (%)**								
	Baseline	45.0 (0.02)		43.0 (0.02)				
	3-months follow-up	36.1 (0.05)	.025	39.5 (0.05)	.431	1.03 (1, 133)	.311	0.67 (0.31 to 1.45)
**Polydrug**^c^ **prevalence (%)**								
	Baseline	42.9 (0.02)		40.1 (0.02)				
	3-months follow-up	31.3 (0.05)	.012	36.8 (0.05)	.235	2.22 (1, 163)	.138	0.57 (0.27 to 1.20)

^a^Based on (logistic) general linear mixed model with group as fixed factor, follow-up values as outcomes, and baseline scores, country, and parental educational attainment as covariates.

^b^Valid follow-up data for n=211 trial participants.

^c^Combined use of alcohol and any illegal drug in past 30 days.

**Table 6:** Intervention effects on primary and (continuous) secondary outcomes (EM-imputed dataset).

Outcomes after 3 months		WISEteens group (n=715)			Control group (n=734)			Between-group differences			
		Mean (SD)	Change from baseline, adjusted mean (95% CI)	*P*	Mean (SD)	Change from baseline, adjusted mean (95% CI)	*P*	Adjusted mean (95% CI)	*F*(df)	*P*	*d*
**Index alcohol use**^b^											
	Baseline	5.24 (2.71)			5.25 (2.78)						
	3-months follow-up	4.72 (1.58)	-0.63 (-0.73 to -0.52)	<.001	4.82 (1.52)	-0.49 (-0.60 to -0.39)	<.001	0.13 (0.02 to 0.25)	5.23 (1, 1329)	.022	.04
**Drinking frequency**^c^											
	Baseline	1.98 (0.81)			1.93 (0.90)						
	3-months follow-up	1.75 (0.47)	-0.24 (-0.27 to -0.20)	<.001	1.76 (0.46)	-0.20 (-0.24 to -0.17)	<.001	0.03 (-0.003 to -0.07)	3.21 (1, 1329)	.073	.11
**Binge drinking frequency**^c^											
	Baseline	1.54 (0.99)			1.58 (1.02)						
	3-months follow-up	1.39 (0.50)	-0.20 (-0.24 to -0.16)	<.001	1.42 (0.47)	-0.16 (-0.20 to -0.12)	<.001	0.03 (-0.01 to -0.08)	2.30 (1, 1329)	.130	.01
**Drinking quantity**^c^											
	Baseline	1.74 (1.46)			1.75 (1.49)						
	3-months follow-up	1.64 (0.77)	-0.15 (-0.20 to -0.10)	<.001	1.71 (0.77)	-0.08 (-0.14 to -0.03)	.001	0.07 (0.01 to 0.12)	5.33 (1, 1329)	.021	.05
**Illegal drug use frequency**^d^											
	Baseline	0.84 (1.15)			0.76 (1.08)						
	3-months follow-up	0.70 (0.76)	-0.12 (-0.15 to -0.08)	<.001	0.67 (0.71)	-0.11 (-0.14 to -0.08)	<.001	0.01 (-0.3 to 0.04)	0.18 (1, 1329)	.670	.04

^a^Based on linear mixed model with group as fixed factor, changes from baseline as outcomes, and baseline scores, country, and parental educational attainment as covariates for continuous outcomes. Cohen’s d calculated by subtracting the average difference score between pretest and posttest of the control group from the corresponding difference score of the intervention group and dividing the result by the pooled standard deviation of the baseline scores.

^b^Adapted AUDIT-C index score (primary outcome).

^c^Adapted AUDIT-C indicators, scores ranging from 0-4 with higher scores indicating more severe drinking.

^d^Scores ranging from 0-4 with higher scores indicating more frequent illegal drug use.

**Table 7:** Intervention effects^a^ on binary secondary outcomes (EM-imputed dataset^b^).

Outcomes after 3-months		WISEteens group (n=715)		Control group (n=734)		Between-group differences		
		% (SE)	*P*	% (SE)	*P*			
						*F*(df)	*P*	OR (95% CI)
**Illegal drug use**p **revalence (%)**								
	Baseline	49.8 (0.02)		49.6 (0.02)				
	3-months follow-up	41.7 (0.02)	<.001	39.8 (0.02)	<.001	1.30 (1, 1446)	.254	1.22 (0.87 to 1.73)
**Polydrug**^c^ **prevalence (%)**								
	Baseline	47.8 (0.02)		46.3 (0.02)				
	3-months follow-up	41.1 (0.02)	<.001	39.8 (0.02)	<.001	0.02 (1, 1446)	.888	1.03 (0.73 to 1.44)

^a^Based on (logistic) general linear mixed model with group as fixed factor, follow-up values as outcomes, and baseline scores, country and parental educational attainment as covariates.

^b^The binary imputed prevalence outcomes (illegal drug use and polydrug use), a real number between 0 and 1 was transformed back into a dichotomous variable by rounding off to two positions behind the decimal point.

^c^Combined use of alcohol and any illegal drug in past 30 days.

## Discussion

### Principal Findings

The purpose of this study was to test the effectiveness of a fully automated Web-based screening and brief motivational intervention targeting adolescents with at-risk substance use in Europe.

We found that self-reported risk drinking as measured by a drinking index (ie, drinking frequency, frequency of binge drinking, and typical quantity of drinks) was significantly reduced for participants in the intervention group. The effect on the primary alcohol use outcome was consistent across imputation and non-imputation (“completers”) based analyses but accented in the non-imputed data analysis, even though statistical power was low for 3-month effects due to large loss to follow-up assessment. Secondary analyses using the three drinking indicators as separate outcomes revealed statistically significant mean differences at follow-up in favor of the WISEteens intervention group for drinking frequency and binge drinking frequency but not quantity when missing follow-up data was not imputed. In contrast, analyses using an EM-imputed dataset revealed drinking quantity as the only significant secondary effect. For illegal drug use or polydrug use, there were no significant intervention effects.

The effect sizes obtained in this study are small but match those summarized in recent systematic reviews for fully automated interventions for young adults [32] and meta-analyses for single session interventions [76, 77]. Moreover, they correspond with effect sizes reported for face-to-face brief interventions for youth who use alcohol and other drugs [17, 78, 79] and indicate that expected effects of MI-based interventions on substance use may indeed be small [41] but can be relevant when a large population can be reached. Overall, there are currently few Web-based interventions targeting adolescents, which limits direct comparisons to prior studies. However, our study contradicts results from one recent RCT that tested the effects of a similar intervention (What Do You Drink [WDYD]). This trial targeted drinking among young people (15-20 years) with low educational background in the Netherlands [80]. While WDYD and WISEteens are comparable in central characteristic (eg, age group, cultural context, single session fully automated delivery mode, intervention duration, applied theory, and outcome measures), there are a number of differences that could account for the divergent effects, such as the school-based study implementation and different follow-up times. Moreover, in the WDYD trial participants with an indication of severe problem drinking at baseline were excluded, while in our study about half of the participants were above the AUDIT-C risk cut-off of 5 points [62] at baseline. Severity of baseline drinking can influence effects of brief motivational interventions with stronger effects among subgroups of heavy drinkers [81].

WISEteens was not effective to address illegal drug use and polydrug use adequately. However, the number of participants with drug and polydrug use was rather low in our sample and meaningful between-group effects might have been undetected due to insufficient statistical power [37]. Note that there were notable decreases in the prevalence rates for polydrug use and illegal drug use in the intervention group, while there were no changes and even slight increases in the control group. There were also no spill-over effects from reduced problem drinking to other substance use, which suggests that effects on targeted outcomes may not translate to untargeted outcomes [82, 83].

In general, effects from comparable Web-based interventions for illegal drugs are typically smaller than for alcohol [32]. Moreover, the main hypothesis and focus of the intervention was on drinking. Some intervention elements, such as decisional balance, importance, and confidence ruler and advice for risk situations were available for alcohol per default and participants with other drug use were explicitly encouraged to take the exercises as templates for use of other substances. Thus, the lack of positive outcomes may also be the result of limited specific intervention content for drugs other than alcohol, which may be a limitation. This notwithstanding, the lack of significant effects in our study corresponds to previous trials among students that failed to promote positive behavior change [84, 85], although such interventions can in principle be effective in the general population [32, 86].

### Strengths and Limitations

Our study is among the first to report on a targeted fully automated Web-based brief intervention among at-risk adolescents with excessive alcohol drinking and drug use in a randomized controlled trial. From a public health perspective, the significant effect on drinking is relevant. Notwithstanding the often dramatic consequences associated with illegal drug use, alcohol is the most frequently used psycho-active substance during adolescence, alcohol use disorders are among the most prevalent and costly mental disorders in industrialized countries, and prevention is a public health priority [87]. Although the main burden of alcohol-related diseases and injuries becomes apparent in adulthood, it is well documented that early at-risk alcohol use can lead to persistent problems [88,89]. Considering the magnitude of youth with at-risk alcohol use the need for effective targeted prevention may be particularly high in Europe. For example in Germany, hospital admissions due to acute alcohol intoxication have increased substantially in the past years, although the proportion of youth who drink alcohol is decreasing [7]. With this study, we provide initial support for the effectiveness of Web-based brief interventions for adolescents in Europe—an approach that has proven useful in other critical heavy drinking populations, such as college students [31, 33].

The WISEteens intervention was designed to reflect valid face-to-face motivational strategies in a Web-based format. We extended individual feedback techniques typically used in Web-based brief interventions by other MI techniques from face-to-face interventions, such as decisional balance, confidence and importance ruler, and provision of behavioral and regulatory strategies to resist peer pressure [38, 40, 90]. Moreover, we aimed to mimic MI principles by applying a carefully designed and pilot-tested motivational dialogue to reflect possibly “empathic” language, acknowledgement of ambivalent goals, needs for autonomy and self-directedness, and appreciation of change but not necessarily abstinence. Although our results cannot address this issue directly, we assume that this may have contributed to the favorable effects revealed in this study. Certainly we acknowledge the limits in delivering MI-consistent methods such as relational factors in a fully automated format [91]. However, arguably the ability of such interventions to deliver standardized intervention content on a large scale and at low cost may outweigh these limitations.

Finally, even though response to follow-up assessment was very low, screening and intervention were accessed and completed by a reasonably large number of teenagers, which may indicate acceptance and ecological validity in the target group. The intervention completion rate indicates acceptable user engagement in the “real world” [92] and a good balance between the required amount of program exposure and adherence requirements of a single session Web-based delivery format for adolescents [50, 93].

Our study has a number of limitations. First and foremost, results are limited by the higher than expected dropout rate for follow-up assessment, which is a frequent problem in Web-based trials [66, 70]. Dropout might be partly caused by invalid email addresses used by the participants and the fact that the system sent only one email reminder per participant [94]. Even though we detected no serious attrition bias, this may limit the validity of the study findings. Although in case of large dropout, any approach to missing data imputation such as the EM method employed in this study could be compromised. We approached this problem by cross-check analyses using an FIML approach to missing data estimation, which yielded similar and significant results for the primary outcome. Moreover, it must be noted that attrition was mainly a problem for the evaluation (ie, attrition took place between measurement points) and much less for intervention adherence (ie, intervention completion rates). The fact that we were able to identify intervention effects, even with the fewer than required number of participants needed to detect small effects in the evaluation, could be interpreted as an underestimation of intervention effects. This notwithstanding and although the study was performed in four European countries, the results should be taken only under careful consideration and follow-up should be translated to other countries or even to other regions or to other groups of adolescents in the four involved countries. The inconsistent results of the imputed and non-imputed analyses and substantial between-analyses deviations in obtained effect sizes serve as a quantitative indicator of uncertainty in these results due to the substantial amount of missing follow-up data.

Another limitation is reliance on self-reported data in this study, which are often associated with underreporting of alcohol drinking and other drug use [95]. Measures to avoid underreporting were assurance of and advice for confidentiality and a non-judgmental and non-confronting MI style employed throughout the intervention. Moreover in our study, the self-report was given anonymously and without personal contact, which may add to the reliability of self-reported data. Moreover, we relied on a primary outcome that was based on the AUDIT-C, which was developed as a screening tool for harmful alcohol use in adults. Given the adapted reference time of drinking in the past 30 days, it is difficult to interpret the practical significance of the measured changes in outcome scores in response to the intervention. Furthermore, participants were not blinded to the assigned interventions, which is a common limitation in Web-based trials [96].

Finally, given the focus on intervention effects in this study, we have not systematically included recruitment and reach in our analyses although these are important issues for estimating public health impact [97]. Rather, we aimed for a convenience sample from the general population by employing a pragmatic recruitment procedure. However, the incitement by lottery as an incentive for participation may have increased the reach to a higher level than can be expected in implementation outside a research project. While the self-selected nature of our sample again limits generalization, we consider the realistic setting of this trial a significant strength. In fact, apart from the evaluation requirements at baseline, the actual intervention program was equivalent to a potential real world application. We thus feel confident in stating that our study has realistic public health implications.

### Conclusion

Web-based interventions to reduce adolescent at-risk substance use hold promise for accessibility and large scale dissemination but have rarely been tested in randomized controlled trials. Although limited by substantial dropout to follow-up assessment, our findings imply that young adolescents with excessive drinking can benefit from targeted interventions based on MI techniques and counseling style in a fully automated Web-based delivery mode. However, we found no effect on drug use, which calls for further research on effective intervention models, delivery modes, and recruitment strategies.

## Appendix

Multimedia Appendix 1 CONSORT-eHEALTH V 1.6.1.

Multimedia Appendix 2 CRAFFT Screening for at-risk substance use (A CRAFFT score of more than one positive item was inclusion criterion).

Multimedia Appendix 3 Screenshots.

Multimedia Appendix 4 Assumed behavior change model and targeted WISEteens content.

Multimedia Appendix 5 Intervention effects at 3-month follow-up, ITT-analysis based on FIML.

## Abbreviations

AUDIT-C: Alcohol Use Disorder Identification Test Consumption subscale
BAC: blood alcohol concentration
CONSORT: Consolidated Standards of Reporting Trials
EM: expectation maximization
FIML: full information maximum likelihood
ITT: intention-to-treat analyses
LMM: Linear Mixed Model
MI: motivational interviewing
RCT: randomized control trial
WDYD: What Do You Drink
