# Supplementary material for: Effectiveness of a Web-Based Screening and Fully Automated Brief Motivational Intervention for Adolescent Substance Use: A Randomized Controlled Trial
Source: J Med Internet Res. 2016 May 24;18(5):e103. doi: 10.2196/jmir.4643 (PMC4897296; doi:10.2196/jmir.4643)
Supplement: Multimedia Appendix 2 [file jmir_v18i5e103_app2.pdf]

WISEteens ...drink clever!

Dein persönliches Trinkverhalten

Um herauszufinden, ob Wiseteens für dich hilfreich sein kann, beantworte bitte die folgenden Fragen. Das dauert nur ein paar Minuten. Es gibt dabei kein "richtig" oder "falsch", und deine Antworten werden selbstverständlich vertraulich behandelt; antworte deshalb bitte ganz ehrlich. Bitte klicke auf "Nächste Seite", sobald du alle Fragen beantwortet hast.

	JA	NEIN
Bist du schon mal selbst unter Alkohol- oder Drogeneinfluss **Auto** gefahren oder bei jemandem mitgefahren, der unter Alkohol- oder Drogeneinfluss stand?	○	○
Trinkst du manchmal Alkohol oder nimmst du Drogen um zu entspannen, dich besser zu fühlen oder damit du dich unter Freunden und Bekannten wohler fühlst?	○	○
Trinkst du jemals Alkohol oder nimmst Drogen, wenn du **alleine** bist?	○	○
Hast du schon mal **vergessen**, was du gemacht hast, als du Alkohol getrunken oder Drogen genommen hast?	○	○
Haben dir **Familienangehörige** oder **Freunde** schon mal geraten, weniger zu trinken oder weniger Drogen zu nehmen?	○	○
Bist du schon mal in **Schwierigkeiten** geraten, als du Alkohol getrunken oder Drogen genommen hast?	○	○

Nächste Seite

brought to you by Universitätsklinikum Hamburg-Eppendorf | Deutsches Zentrum für Suchtfragen des Kindes- und Jugendalters (DZSKJ)
