# Supplementary material for: Effectiveness of a Web-Based Screening and Fully Automated Brief Motivational Intervention for Adolescent Substance Use: A Randomized Controlled Trial
Source: J Med Internet Res. 2016 May 24;18(5):e103. doi: 10.2196/jmir.4643 (PMC4897296; doi:10.2196/jmir.4643)
Supplement: Multimedia Appendix 3 [file jmir_v18i5e103_app3.pdf]

Multimedia Appendix 3. Screenshots.

Fragen zu deinem Trinkverhalten und Drogengebrauch

Seite 4 von 10
Bitte gib an, welche der folgenden Aussagen deine aktuellen Trinkgewohnheiten am besten beschreibt.

Hast du in den letzten 30 Tagen 5 (4 für Mädchen) oder mehr alkoholische Getränke hintereinander konsumiert? :

- Ja, und ich habe nicht vor, damit aufzuhören 5 oder mehr alkoholische Getränke hintereinander zu trinken.
- Ja, aber ich habe vor, während der nächsten 6 Monate damit aufzuhören 5 oder mehr alkoholische Getränke hintereinander zu trinken.
- Ja, aber ich habe vor, während der nächsten 30 Tage damit aufzuhören 5 oder mehr alkoholische Getränke hintereinander zu trinken.
- Nein, aber ich habe während der letzten 3 Monate 5 oder mehr alkoholische Getränke hintereinander getrunken.
- Nein, und ich habe auch während der letzten 3 Monate niemals 5 oder mehr alkoholische Getränke getrunken.
- Nein, ich habe noch nie 5 oder mehr alkoholische Getränke hintereinander getrunken.

In den letzten 30 Tagen:

Wenn deine Antwort auf die erste Frage "niemals" ist, brauchst du die folgenden Fragen auf dieser Seite über dein Trinkverhalten oder deinen Drogenkonsum innerhalb der **vergangenen 30 Tage** nicht zu beantworten.

	niemals	Ein Mal	2-4 Mal im Monat	2-3 Mal pro Woche	4 Mal oder mehr pro Woche
Wie häufig hast du in den vergangenen 30 Tagen ein alkoholisches Getränk konsumiert?	○	○	○	○	○
Wie häufig hast du in den vergangenen 30 Tagen bei einer Gelegenheit (z.B. auf einer Party) 5 oder mehr (für Mädchen 4 oder mehr) alkoholische Getränke zu dir genommen?	○	○	○	○	○

Wie viele alkoholische Getränke hast du innerhalb der vergangenen 30 Tage an einem typischen Tag, an dem du trinkst, konsumiert?:

0

* ein "Standardgetränk" enthält 10-12g reinen Alkohol. Dies kann genau ein Getränk sein (z.B. ein kleines Bier von 250ml, ein kleines Glas Wein von 125ml oder ein doppelter Schnaps / Korn von 40ml), aber eine Flasche Bier (500ml) oder ein Viertelliter Wein (250ml) zählen als 2 Standardgetränke!

	niemals	Ein Mal	2-4 Mal im Monat	2-3 Mal pro Woche	4 Mal oder mehr pro Woche
Wie häufig hast du in den vergangenen 30 Tagen Drogen außer Alkohol konsumiert?	○	○	○	○	○
Wie häufig hast du in den vergangenen 30 Tagen bei derselben Gelegenheit verschiedene Sorten von Drogen außer Alkohol konsumiert?	○	○	○	○	○

Wie viele Male hast du in den vergangenen 30 Tagen Drogen außer Alkohol genommen, an einem typischen Tag an dem du Drogen konsumierst?:

0

Während der letzten 30 Tage, wie häufig bist du betrunken oder stärker berauscht von Alkohol gewesen?:

0

Nächste Seite >

WISEteens ...drink clever!

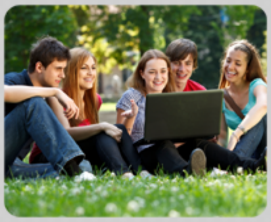

Wiseteens helps you with practical tips to control your alcohol consumption. So that you can enjoy life to the fullest – even without alcohol and drugs.

Danke für deine Antworten, Paul!

Es geht jetzt mit dem zweiten Teil des Wiseteens-Programms weiter.

Wahrscheinlich fragst du dich jetzt, was das alles soll – warum diese ganzen Fragen?

Sie helfen dir, deinen Alkohol- und Drogenkonsum (falls du Drogen nimmst) besser einzuschätzen. So kannst du leicht für dich selbst entscheiden, ob du das okay findest, wie es ist, oder ob du vielleicht etwas daran verändern willst.

Das Ganze läuft so ab:

- Gleich bekommst du ein **individuelles Feedback** zu deinen Antworten.
- Danach bekommst du **praktische Tipps**, die speziell für dich gut passen könnten. Diese Tipps sind dafür gedacht, dass du in bestimmten Situationen (zum Beispiel auf Partys oder wenn du Stress hast) dein Trinkverhalten im Griff behalten kannst – und trotzdem die Dinge schaffst, die du dir vorgenommen hast.

Such' dir als Nächstes eine von den Antworten unten aus, dann geht es weiter. Das Ganze dauert insgesamt ungefähr 15 bis 20 Minuten.

- ○ Was heißt "individuelles Feedback"?
- ○ Okay, dann los.
- ◉ Wer entscheidet eigentlich, an wen die iPads gehen?

Ok

Impressum Logout

brought to you by Universitätsklinikum Hamburg-Eppendorf | Deutsches Zentrum für Suchtfragen des Kindes- und Jugendalters (DZSKJ)

WISEteens ...drink clever!

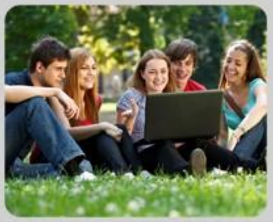

Wiseteens helps you with practical tips to control your alcohol consumption. So that you can enjoy life to the fullest – even without alcohol and drugs.

Die iPads werden unter allen verlost, die hier bis zum Schluss mitmachen. Ausgelost wird das nach dem Zufallsprinzip – jeder hat die gleiche Chance.

Schauen wir uns zunächst mal an, wie viel du zurzeit trinkst. Dafür wurden deine Antworten von vorhin zusammengezählt und ausgewertet. Für dich ergibt sich daraus:

> ! **Nach deinen Angaben lag dein Alkoholkonsum im letzten Jahr in einem wirklich hohen und riskanten Bereich.**

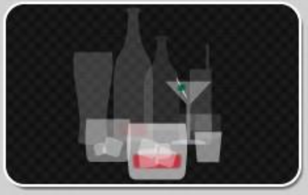

Wie viel du trinkst, ist natürlich deine eigene Sache. Deine Antworten weisen jedoch auf einen gefährlichen Alkoholkonsum hin...

Alkohol scheint eine bedeutsame Rolle in deinem Leben zu spielen. Vielleicht hast du dir noch gar keine Gedanken darüber gemacht, wie es eigentlich dazu kam? Es könnte aber hilfreich sein, wenn du dir mal bewusst machst, warum du so viel trinkst.

ⓘ Wenn du mehr über Alkoholkonsum erfahren möchtest, klick bitte auf den blauen Button.

- ○ Mir war das gar nicht bewusst, dass ich *so* viel trinke...
- ○ Was soll's! Andere trinken genauso viel, und ich fühle mich gut dabei.
- ○ Manchmal passieren mir peinliche Sachen im Rausch, das ist mir schon unangenehm...

Ok

brought to you by Universitätsklinikum Hamburg-Eppendorf | Deutsches Zentrum für Suchtfragen des Kindes- und Jugendalters (DZSKJ)

WISEteens ...drink clever!

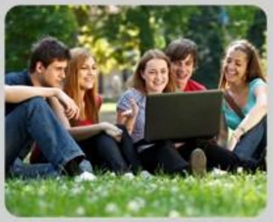

Wiseteens helps you with practical tips to control your alcohol consumption. So that you can enjoy life to the fullest – even without alcohol and drugs.

Der Gedanke ist verständlich, und in deinem Alter ist es auch ganz normal, dass man das nicht als Problem sieht, selbst wenn man mal mehr getrunken hat. Trotzdem könnte es sich lohnen, einmal genauer darüber nachzudenken...

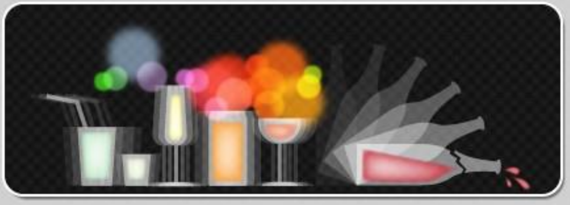

Dein Promillewert
Vorhin hast du in deinen Antworten eine Situation beschrieben, bei der du viel getrunken hattest. Vielleicht weißt du noch, wann und wo das war - zum Beispiel auf einer Party oder beim Weggehen. Hier kommt dazu mal eine Auswertung, die dir zeigen soll, wie viel "Promille" du dabei in etwa hattest.

Du hattest bei dieser Gelegenheit wahrscheinlich einen Promillewert von

von 4,0 oder mehr Promille.

Dieser Wert ist extrem hoch; man spricht dabei nicht mehr von "Rausch", sondern von einer "lebensbedrohlichen Lähmung".

Wenn man so viel getrunken hat, hat man eine lebensbedrohliche Alkoholvergiftung, die schnellst möglich medizinisch behandelt werden muss. Die Folgen einer solchen Alkoholvergiftung sind Bewusstlosigkeit und die Gefahr, sehr stark zu unterkühlen. Eine schwache Atmung oder sogar ein Aussetzten der Atmung und Probleme mit deinem Herzschlag können dich in ein Koma fallen lassen und zum Tod führen.

ⓘ Wenn du mehr über Promillewerte und deren Auswirkungen erfahren möchtest oder wissen willst, wie dein Promillewert berechnet worden ist, dann klicke einfach auf den blauen Info-Button.

- ○ Das war wirklich ein krasser Rausch, meine Freunde haben's mir hinterher erzählt...
- ○ Das war die absolute Ausnahme! Normalerweise trinke ich nicht so viel.
- ○ Ich bin oft so besoffen, das macht mir Spaß und gehört zum Leben dazu.

Ok

brought to you by Universitätsklinikum Hamburg-Eppendorf | Deutsches Zentrum für Suchtfragen des Kindes- und Jugendalters (DZSKJ)

WISEteens ...drink clever!

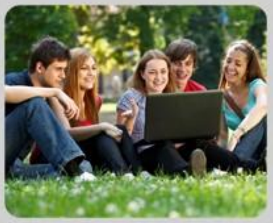

Wiseteens helps you with practical tips to control your alcohol consumption. So that you can enjoy life to the fullest – even without alcohol and drugs.

Das war es bestimmt. Und vielleicht war das ja auch wirklich nicht besonders angenehm - man kann sich denken, dass du dich wirklich nicht gut gefühlt hast dabei...

Anfangs haben wir dich gefragt, wie oft du trinkst und auch, was du glaubst, wie oft ein typischer Jugendlicher in deinem Alter wohl so trinkt. Unten siehst du diese Antworten noch mal. Gleichzeitig kannst du ablesen, wie oft andere Jugendliche deines Alters *wirklich* trinken.

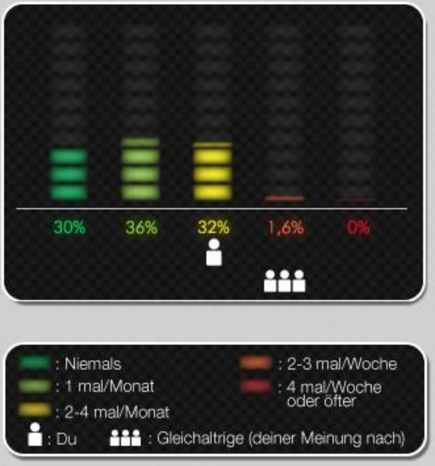

- ○ Alles klar, genauso hab ich mir das schon gedacht.
- ○ Wow, das hätte ich nicht gedacht...
- ◉ Eigentlich interessiert mich mehr, wie *viel* andere bei einer Gelegenheit trinken...

Ok

Impressum Logout

brought to you by Universitätsklinikum Hamburg-Eppendorf | Deutsches Zentrum für Suchtfragen des Kindes- und Jugendalters (DZSKJ)

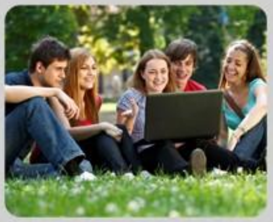

Wiseteens helps you with practical tips to control your alcohol consumption. So that you can enjoy life to the fullest – even without alcohol and drugs.

Dass Drogen Spaß machen können, wollen wir gar nicht bestreiten - schließlich würde sie ja sonst niemand nehmen. Genauso wahr ist es allerdings, dass sie gefährlich für deine Gesundheit sein können. Die Entscheidung, ob du Drogen nimmst oder nicht, liegt bei dir.

Bisher ging es sehr ausführlich um deinen Konsum von Alkohol und/oder Drogen. Du hast eine ganz auf deine Angaben zugeschnittene Rückmeldung erhalten, die dir dabei helfen soll, deine Trinkgewohnheiten und möglicherweise auch deinen Drogenkonsum besser einschätzen zu können. Es würde uns also freuen, wenn du dich durch Wiseteens besser informiert fühlst.

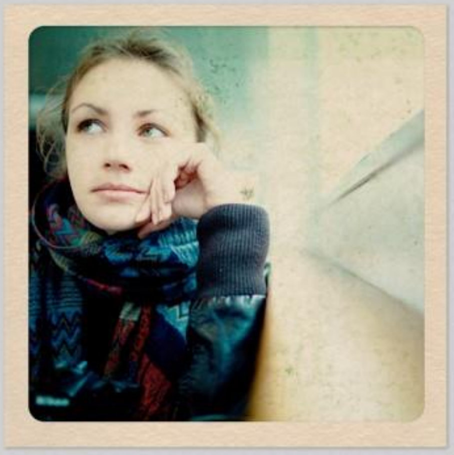

Als Nächstes geht es um deine Sicht zum Thema Alkohol und Drogen. Dazu wollen wir dir ein paar kurze Übungen anbieten, die dir u.a. dabei helfen können, dir einmal die Vor- und Nachteile von Alkohol- und Drogenkonsum bewusst zu machen und zwar aus deiner ganz persönlichen Perspektive. Wenn du einmal über deine Trinkgewohnheiten nachdenkst:

Wie *wichtig* fändest du es, deinen aktuellen Alkoholkonsum zu reduzieren und weniger zu trinken?

Entscheide dich bitte für eine der Zahlen von 0 bis 10.

- **0 bedeutet dabei:** Es ist dir völlig unwichtig, weniger zu trinken
- **10 würde bedeuten:** Es ist dir sehr wichtig, weniger Alkohol zu trinken

- ○ 0
- ○ 1
- ○ 2
- ○ 3
- ○ 4
- ○ 5
- ○ 6
- ○ 7
- ○ 8
- ○ 9
- ○ 10

Ok

WISEteens ...drink clever!

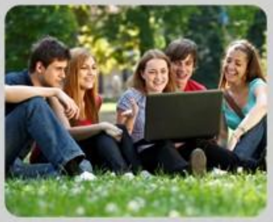

Wiseteens helps you with practical tips to control your alcohol consumption. So that you can enjoy life to the fullest – even without alcohol and drugs.

Alles klar, danke für deine Einschätzung. Momentan ist es also eher **weniger wichtig für dich**, deinen Alkoholkonsum einzuschränken. Vielen Dank für deine ehrliche Einschätzung.

Vielleicht lohnt es sich dennoch für dich, dir folgende Fragen zu stellen:

- Wobei hilft mir mein aktueller Alkoholkonsum?
- Aus welchen Gründen trinke ich denn Alkohol?
- Welche „Schattenseiten" hat mein aktueller Alkoholkonsum?
- Was spricht für mich dagegen, in der Zukunft weniger zu trinken?

Und auch einmal andersherum:

- Was spricht für mich dafür, in der Zukunft weniger zu trinken oder gar damit aufzuhören?

Manche Leute können solche Fragen schnell beantworten. Die meisten brauchen jedoch Zeit, da sie vermutlich nie bewusst darüber nachgedacht haben. Daher, lass dir Zeit!

Der Hintergrund dieser kleinen Frage-Übung ist wieder der gleiche wie bisher: Sie soll dir helfen, mehr über dich selbst und dein Trinkverhalten herauszufinden. Die gleichen Fragen kannst du dir übrigens auch zum Thema "Drogen" stellen.

- ○ Verstehe... um ehrlich zu sein, ich hab' mir wirklich noch nie bewusst Gedanken über diese Fragen gemacht - bisher war alles einfach nur so, wie es ist...
- ○ Hm... irgendwie bringt mich das nicht richtig weiter... geht das auch ein bisschen konkreter?
- ○ Das ist lahm, ich schalte gleich ab...
- ○ Ich trinke eben manchmal... warum der ganze Aufriss hier?

Ok

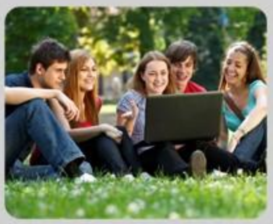

Wiseteens helps you with practical tips to control your alcohol consumption. So that you can enjoy life to the fullest – even without alcohol and drugs.

Vielen Dank. Wir versuchen mal, deine Antworten zusammenzufassen und zu beschreiben, was sie für dein Trinkverhalten bedeuten könnten.

Du siehst anscheinend momentan weder besonders viele Vorteile noch Nachteile an deinem Trinkverhalten – irgendwie scheint sich das bei dir gerade die Waage zu halten.

Ein bisschen anders scheinst du das aber zu sehen, wenn du an die Zukunft denkst. Offenbar hast du den Eindruck: *"In Zukunft weniger zu trinken – das würde mir nichts bringen"*. Jedenfalls überwiegen für dich derzeit eher die Nachteile weniger zu trinken. Kommt das ungefähr hin? Die Grafik hier hilft dir dabei, deine Auswahl zu deuten. Das funktioniert so:

- In jedem der vier Felder befinden sich ein paar wenige oder ziemlich viele bunte Kreise.
- Ein paar **wenige Kreise** bedeuten: Die Gründe, die in dieses Feld passen würden - zum Beispiel die "Vorteile deines aktuellen Alkoholkonsums" - spielen für dich keine wichtige Rolle.
- Sind es dagegen ziemlich **viele Kreise**, bedeutet das: Dieses Feld findest du schon eher wichtig - zum Beispiel die "Vorteile, weniger Alkohol zu trinken".

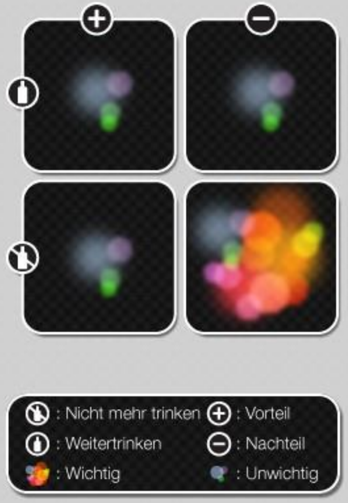

Übrigens:
Diese Übung kannst du auch gerne dann anwenden und für dich nutzen, wenn es um deinen Drogenkonsum geht.

- ( ) Ich finde, das passt für mich ganz gut...
- ( ) Irgendwie sagt mir diese Grafik gar nichts...
- ( ) Also, irgendwie stimmt diese Einschätzung nicht für mich...

Ok
