# Supplementary figures and images for: Effectiveness of a Web-Based Screening and Fully Automated Brief Motivational Intervention for Adolescent Substance Use: A Randomized Controlled Trial
Source: J Med Internet Res. 2016 May 24;18(5):e103. doi: 10.2196/jmir.4643 (PMC4897296; doi:10.2196/jmir.4643)

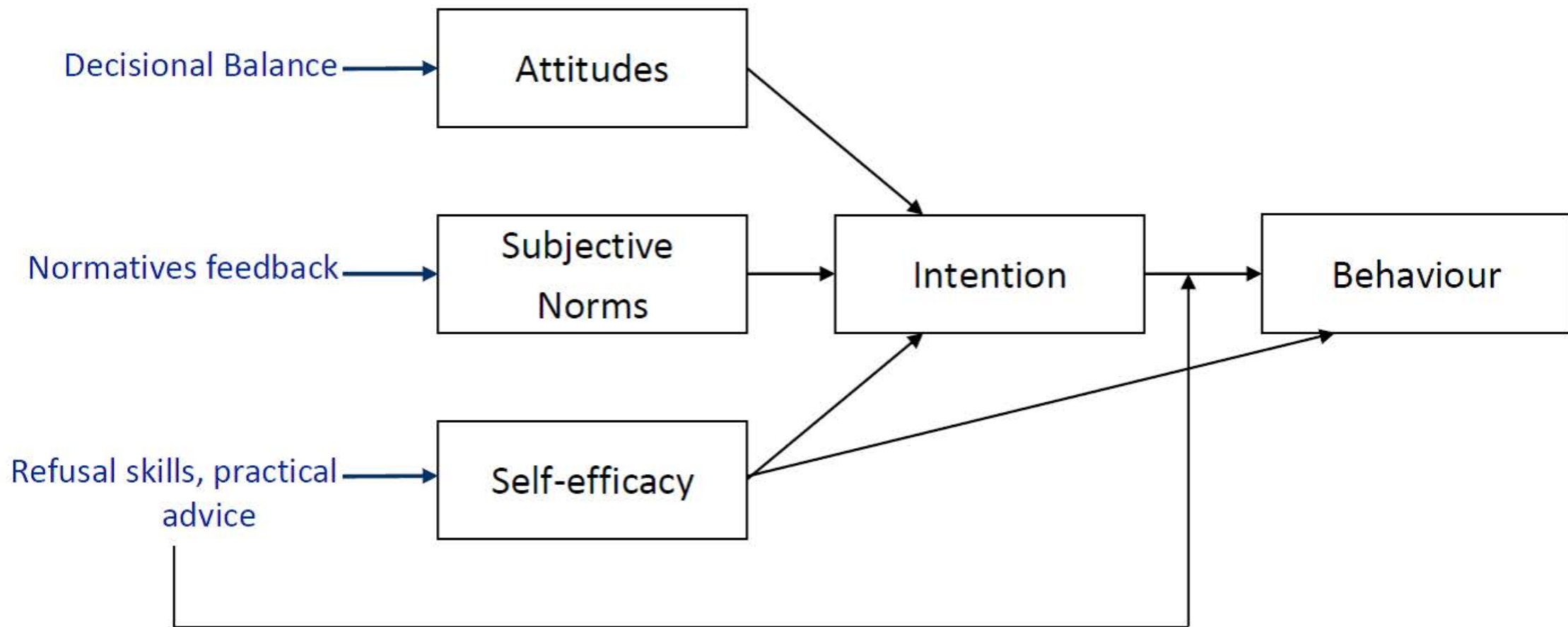

Supplement: Multimedia Appendix 4 [file jmir_v18i5e103_app4.pdf]
