# Supplementary material for: Effectiveness of a Web-Based Screening and Fully Automated Brief Motivational Intervention for Adolescent Substance Use: A Randomized Controlled Trial
Source: J Med Internet Res. 2016 May 24;18(5):e103. doi: 10.2196/jmir.4643 (PMC4897296; doi:10.2196/jmir.4643)
Supplement: Multimedia Appendix 5 [file jmir_v18i5e103_app5.pdf]

Multimedia appendix: Intervention effects at 3-month follow-up, ITT-analysis based on FIML (N=1449) [a]

	B (β)	*SE*	*P*
Outcome Variable:			
Alcohol:			
AUDIT-C based drinking index	-0.72 (-0.13)	0.37	.046*
Drinking Frequency	-0.13 (-0.08)	0.11	.230
Binge drinking Frequency	-0.25 (-0.14)	0.18	.059
Drinking Quantity	-0.22 (-0.08)	0.18	.209
Illegal drug use			
Frequency	-0.03 (-0.04)	0.13	.834

Note: All outcomes concern last 30 days. All analyses are adjusted for baseline scores, country of residence and parental educational attainment. Missing follow-up outcome values were estimated with FIML.

[a]AMOS does not provide confidence intervals for parameter estimates.
